# Supplementary material for: Causal Relationship between Aging and Anorexia Nervosa: A White-Matter-Microstructure-Mediated Mendelian Randomization Analysis
Source: Biomedicines. 2024 Aug 16;12(8):1874. doi: 10.3390/biomedicines12081874 (PMC11351342; doi:10.3390/biomedicines12081874)
Supplement: Supplementary file 1 [file biomedicines-12-01874-s001.zip › Supplementary Figure/Figure S9/Leaveoneout plot of MR analysis for ubm-b-1633 to AN.pdf]

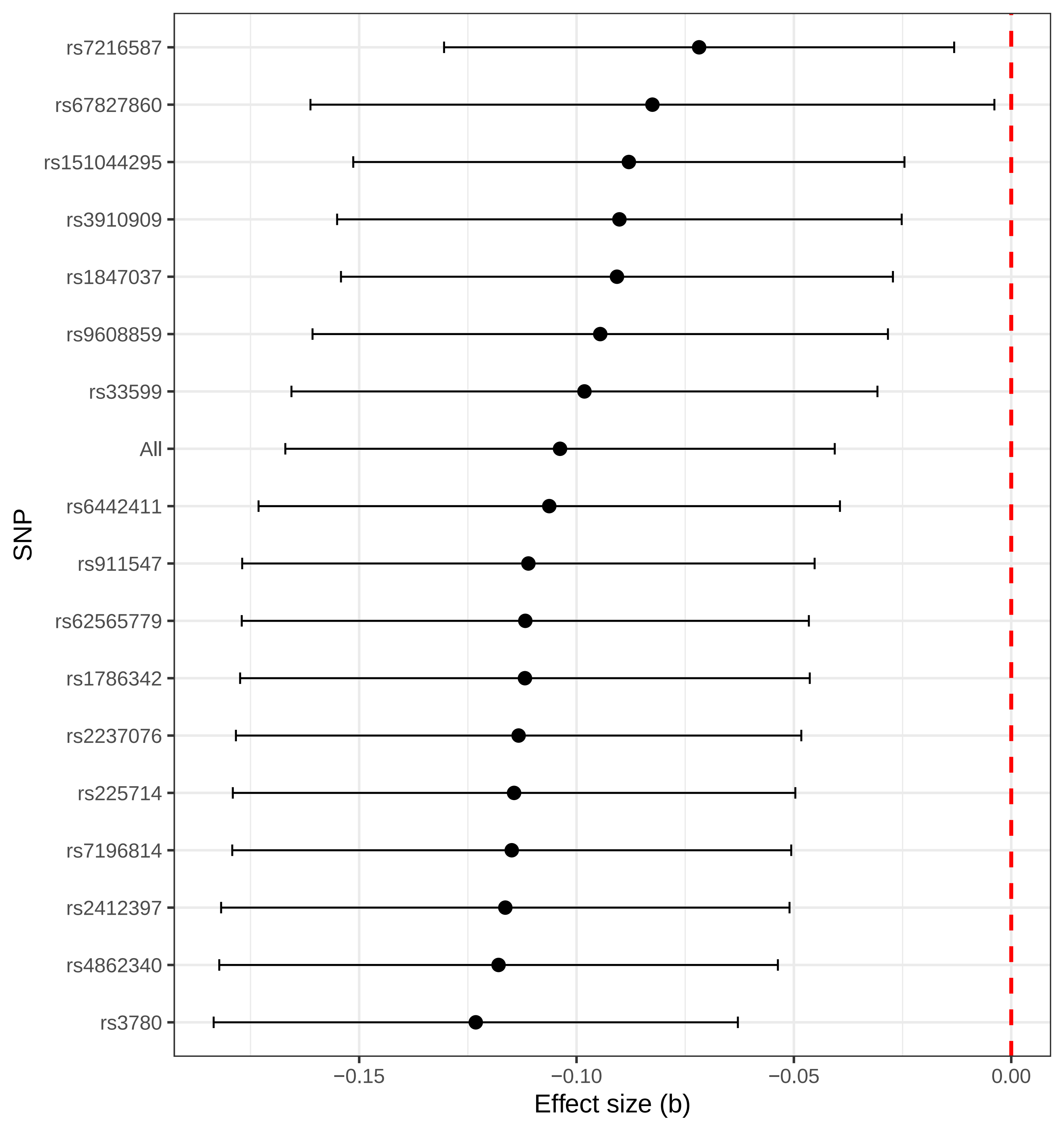
rs7216587
rs67827860
rs151044295
rs3910909
rs1847037
rs9608859
rs33599
All
rs6442411
rs911547
rs62565779
rs1786342
rs2237076
rs225714
rs7196814
rs2412397
rs4862340
rs3780
SNP
−0.15
−0.10
−0.05
0.00
Effect size (b)
